# Measuring child survival for the Millennium Development Goals in Africa: what have we learned and what more is needed to evaluate the Sustainable Development Goals?

**DOI:** 10.1080/16549716.2020.1732668

**Published:** 2020-03-02

**Authors:** Marie A. Brault, Kasonde Mwinga, Aaron M. Kipp, Stephen B. Kennedy, Margaret Maimbolwa, Precious Moyo, Kenneth Ngure, Connie A. Haley, Sten H. Vermund

**Affiliations:** a Department of Social and Behavioral Sciences, Yale School of Public Health, New Haven, CT, USA; b Rwanda Country Office, World Health Organization, Kigali, Rwanda (Formerly, WHO African Regional Office, Brazzaville, Congo); c Vanderbilt Institute for Global Health, Vanderbilt University, Nashville, TN, USA; d Department of Medicine, Division of Epidemiology, Vanderbilt University Medical Center, Nashville, TN, USA; e University of Liberia-Pacific Institute for Research & Evaluation (UL-PIRE) Africa Center, University of Liberia, Monrovia, Liberia; f Department of Nursing Sciences, University of Zambia School of Medicine, Lusaka, Zambia; g Collaborative Research Program, University of Zimbabwe–University of California, San Francisco, Harare, Zimbabwe; h Department of Community Health, Jomo Kenyatta University of Agriculture and Technology, Nairobi, Kenya; i Division of Infectious Diseases and Global Medicine, University of Florida, Gainesville, FL, USA; j Office of the Dean, Yale School of Public Health, New Haven, CT, USA

**Keywords:** Africa, child health, leadership, health services, Sustainable Development Goals, qualitative research

## Abstract

Reducing child mortality is a key global health challenge. We examined reasons for greater or lesser success in meeting under-five mortality rate reductions, i.e. Millennium Development Goal #4, between 1990 and 2015 in Sub-Saharan Africa where child mortality remains high. We first examined factors associated with child mortality from all World Health Organization African Region nations during the Millennium Development Goal period. This analysis was followed by case studies of the facilitators and barriers to Millennium Development Goal #4 in four countries – Kenya, Liberia, Zambia, and Zimbabwe. Quantitative indicators, policy documents, and qualitative interviews and focus groups were collected from each country to examine factors within and across countries related to child mortality. We found familiar themes that highlighted the need for both specific services (e.g. primary care access, emergency obstetric and neonatal care) and general management (e.g. strong health governance and leadership, increasing community health workers, quality of care). We also identified methodological opportunities and challenges to assessing progress in child health, which can provide insights to similar efforts during the Sustainable Development Goal period. Specifically, it is important for countries to adapt general international goals and measurements to their national context, considering baseline mortality rates and health information systems, to develop country-specific goals. It will also be critical to develop more rigorous measurement tools and indicators to accurately characterize maternal, neonatal, and child health systems, particularly in the area of governance and leadership. Valuable lessons can be learned from Millennium Development Goal successes and failures, as well as how they are evaluated. As countries seek to lower child mortality further during the Sustainable Development Goal period, it will be necessary to prioritize and support countries in quantitative and qualitative data collection to assess and contextualize progress, identifying areas needing improvement.

## Background

Millennium Development Goal 4 (MDG4) sought to reduce under-five mortality (U5M) rates by two-thirds between 1990 and 2015 [1]. Although the global U5M rate declined by over 50%, MDG4 was not achieved worldwide. While Sub-Saharan Africa (SSA) has the highest child mortality rates of any region in the world [2], 12 of the 47 countries (26%) in the World Health Organization (WHO) African region did achieve MDG4 [2]. Some of these success stories are detailed elsewhere [3–6]. Our team elucidated barriers and facilitators to achieving MDG4 in SSA. We also collected and analyzed detailed data from two countries that achieved the MDG4 goal (Liberia and Zambia) and two countries that did not (Kenya and Zimbabwe). In this work, we used existing local, national, and international data from agencies, governments, and non-governmental organizations (NGOs), and qualitative interviews with key informants, to explore why some countries made faster progress than others.

We used data from the 46 member countries of the WHO African region, and confronted substantial methodological challenges, particularly regarding incomplete or unreliable data. This forced choices as to how to deploy data fairly and effectively. In this paper, we present our MDG evaluation insights based on this data-intensive experience, with the hope that these insights might contribute to assessments of progress towards the Sustainable Development Goals (SDGs). Specifically, we note the challenges in obtaining reliable and timely data for monitoring progress, and pose the question: what data need to be collected to enable robust evaluations of child survival in SSA during the SDGs? In forecasting these data needs, we hope that countries and the WHO will be better positioned to evaluate progress towards SDGs and to evaluate the factors contributing to success or failure to reach SDGs.

## A summary of key findings

Our work examined socioeconomic and health factors that might be associated with more rapid reductions in U5M from 2000 to 2013 from the 46 nations that were within the WHO African region during that period (excluding South Sudan which joined in 2011 and had limited data) [7]. We assessed the annual rate of change of 70 factors and their association with the annual rate of reduction (ARR) of U5M rates via linear regression models. The overall trends were favorable for most of these factors in most countries, especially for economic or technological development and external financing metrics. However, missing data were common; only 41 of the 70 factors were reported from enough countries to conduct meaningful analyses. Of these 41 factors, only a few had a significant association with higher U5M ARR, adjusting for potential confounders. These associations were especially notable: a positive association of treatment for acute respiratory infection and increasing health expenditure relative to gross domestic product. An inverse association between changes in maternal mortality and U5M ARRs indicated that more rapid declines in maternal mortality were associated with larger ARRs. Despite the dearth of significant predictors and the prevalence of missing data, we concluded that improvements in sociodemographic, maternal health, and governance and financing factors were likely associated with larger U5M ARR [7].

Given the interpretive complexity of the ecological study of factors predicting U5M at the country level, we initiated four country case studies to seek specific factors that may have influenced child survival within each country. Due to the limitations of health information systems in these countries [8] and quantitative indicator data to provide more nuanced understanding [9], we triangulated data from a policy review, qualitative key informant interviews with stakeholders working at multiple levels of the health system, and focus groups with women accessing services for their children (see supplemental file for interview and focus group discussion guides). Countries were chosen based on their U5M ARR between 1990 and 2011 (data available at the study’s onset) and their health ministries’ willingness to participate [10–14]. Two nations, Liberia and Zambia, were selected by our team because they were on track to achieve MDG4 in 2013 when the study was initiated (both countries did, ultimately, meet MDG4). In Liberia, we found the following factors to be critical to their reduction of U5M: national prioritization of MNCH after the civil war combined with continuous review and updates to key strategies; implementation of integrated services in facilities that expanded intervention access and promoted intersectoral collaboration; and use of outreach campaigns, community health workers and trained traditional midwives to expand access to care and improve referrals [15]. In Zambia, we found qualitative evidence of the benefits of Zambia’s ongoing and long-term national health reform focused on ensuring universal access to MNCH care, specifically through strong community health strategies and service delivery approaches as well as positive and well-coordinated relationships with external partners [14].

The other two nations, Kenya and Zimbabwe, were selected because they were not on track to meet MDG4. We found that Kenya’s child survival efforts were hindered by pervasive inequities in access and utilization of MNCH care [11]. However, Kenya has worked to expand infrastructure and MNCH interventions, especially for neonates, and also tried to expand community level service delivery [11,12]. Many of Kenya’s new efforts appeared at the end of the MDG period, and the impact of these efforts should be assessed in the current SDG period. In Zimbabwe, we found that the country’s economic crisis and precipitously declining family incomes reduced both the availability of trained health workers and the development of legislation to address this shortage [13]. These elements, in turn, limited the availability, quality, and utilization of MNCH interventions. Zimbabwe, like Kenya, more actively attempted to address these challenges towards the end of the MDG period, and the impacts of these efforts is worth assessing.

Finally, we compared elements of MNCH governance and leadership across the four countries in an integrated review, as these were elements that emerged from the individual studies [16]. We reviewed the findings and conducted a secondary analysis of the qualitative data from the four country case studies to highlight factors related to health governance and leadership that can contribute to countries seeking to extend their MDG progress, as well as countries planning to renew and improve their efforts in the SDG period. From this study, we found three components of health governance and leadership to be important (Table 1) [1]: establishing child survival as a national priority [2]; involving multi-sectoral stakeholders and collaborations to participate in MNCH planning and implementation; and [3] creating and maintaining accountability through a ‘monitor-review-act’ approach. Without this overarching guidance, individual MNCH interventions remain fragmented and under-resourced [17–20]. Although some countries were able to effectively implement the monitor-review-act approach, greater investment in accurate, responsive, and integrated monitoring and evaluation systems will be needed to assess progress towards the more complicated and ambitious SDGs [8,21–24].

**Table 1.** Health management, governance, and leadership differences between MDG 4 progressing (Liberia, Zambia) and non-progressing (Kenya, Zimbabwe) countries, 2000–2013. Adapted from [16]

	Progressed well		Did not progress well	
	Liberia	Zambia	Kenya	Zimbabwe
**Prioritization and Support of Child Survival**				
Clear political support for child survival investments	+	+	+/−	+/−
Current policy framework highlighting child survival action	+	+	+	−
Policies and strategies implemented towards child survival	+	+	−	−
Concurrent national policy focus on health, social welfare, development	+	+	−	−
Abuja Declaration target* met during study	+	+	−	−
**Collaboration, Coordination and Inclusion**				
Donors aligned with national priorities	+	+	−	−
Collaborative strategic planning with partners/stakeholders	+	+	−	−
Coordination/collaboration between health and other sectors	+	+	−	−
Coordination and sharing resources among different health programs	+	+	−	−
Coordination of MNCH services across health system levels	+	+	−	−
Integrate packages of health services at point of care	+	+	−	−
Decentralization of decision-making and resource allocation	+	+	+/−	−
Beneficiaries included in strategic planning through community input	+	+	−	−
**Accountability**				
Clear roles, responsibilities, and expectations	+	+	+/−	−
Updated, effective Health Management Information Systems	+	+	−	−
Consistent data collection and reporting at all health system levels	+	+	−	−
Ongoing monitoring and evaluation of health programs and interventions	+	+	−	−
Specifically monitoring of progress towards Millennium Development Goal 4**	+	+	+/−	+/−
Data-driven planning and decision making responsive to population needs	+	+	−	−
Local involvement through community planning boards and committees	+	+	−	−

+ Clear activity, policy, participation, and/or implementation of an element in the defined area during the study period.

− Lack of effective engagement of this element, including planning, but not implementing policy/action during the study period.

+/− Ambiguous activity, policy, participation, and/or implementation of elements in this area, or early failures with later progress.

N/A = not available; MNCH = Maternal, neonatal, and child health

* See https://www.who.int/healthsystems/publications/abuja_report_aug_2011.pdf?ua=1, accessed 18 April 2019.

** Child survival goal: See https://www.who.int/topics/millennium_development_goals/child_mortality/en/, accessed 18 April 2019.

## Limitations

**Figure 1.**
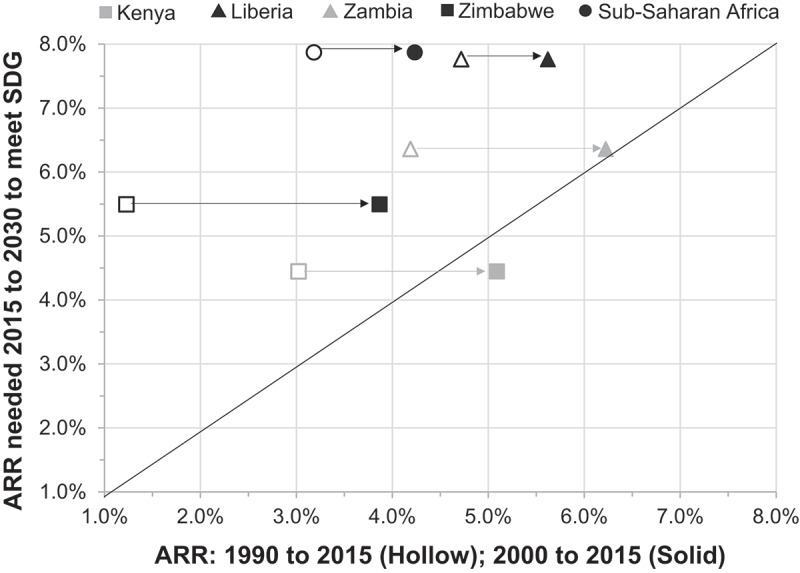
Recent annual rate of reduction (ARR) of under-five mortality compared to the ARR needed to meet the Sustainable Development Goal of 25 deaths per 1,000 for two countries that met their Millennium Development Goal (triangle), two countries that did not meet their MDG (square), and the entire Sub-Saharan Africa region (circle; did not meet MDG Goal)

Elements of our mixed-methods study have limitations, and these limitations may extend to other efforts to evaluate and compare progress towards international goals. First, the 1990 reference point was only established as the beginning of the MDG period in 2001, when the MDGs were formally presented as a policy agenda. Thus, analytic problems emerge with mismeasurement and make it difficult to rely on pre-2000 trends to evaluate the MDG period [3,25]. Measuring achievement by including pre-2000 measures led to our classification of countries as ‘on-track’ or ‘not on-track.’ However, an examination of post-2000 trends suggests that these countries may not have been as different from each other as originally thought. Some countries with greater difficulties and higher mortality rates appear to have made greater acceleration and gains on MDG4 than countries with more moderate mortality rates (Figure 1) [26]. Indeed, our own regional analysis points out that Kenya and Zimbabwe’s slower declines in U5M may have been shaped, in part, by the difficulties in reducing preventable child deaths when starting from a lower baseline U5M rate than Liberia or Zambia [16]. Also, Kenya accelerated its progress towards the end of the MDG period, while Zimbabwe made progress at an even later time period, creating further challenges in comparing two very different ‘off-track’ countries. Looking towards the SDG of 25 or fewer under-five deaths per 1,000 live births, it may be more difficult for countries with higher mortality rates to reach this goal. Of the four countries examined, Liberia and Zambia will need to sustain a higher rate of reduction to meet the SDG (Figure 1), despite successfully meeting their MDG4 targets. The countries examined in this study are all currently collecting new Demographic and Health Surveys (DHS) data, which will provide updated values and a clearer picture of how these countries are faring. The DHS are nationally-representative household surveys that collect data on a variety of population and health indicators in low- and middle-income countries, and the DHS program provides support in the collection and dissemination of the survey data. It may also be important for countries to collect data on key indicators more frequently (yearly) than permitted by the DHS survey cycle (once every five years).

An additional concern is that country-level analyses of intervention coverage mask inequalities and disparities within countries themselves, whether within a city, across rural-urban communities, or within different rural areas [27,28]. Inadequate attention to these disparities can result in inefficient deployment of resources, limiting further reductions in U5M [29]. The larger the nation, the more potentially misleading a country summary might be, as with Nigeria or South Africa, India or China, Brazil or Mexico. Conclusions may be more robust for smaller and/or more homogenous nations.

The policy review was hindered by lack of comparability between the documents that different countries developed and provided for our study. Further, the overlapping time periods of some documents made it difficult to discern when one policy or strategy ended and the next came into effect. The limitations of the qualitative data are primarily related to generalizability, and have been summarized for each country in the case studies.

## Recommendations and conclusions

We offer some recommendations for evaluators of the SDGs based on this work, including countries as well as their donor, NGO, WHO, and academic partners. It is important to have clarity on the time frame for evaluating countries. The baseline year for comparison should have data on the SDG metric being measured. In addition, generalized international measurements and goals may be less useful than country-specific goals that consider a country’s baseline and inequities. Supplementing quantitative indicator data with qualitative, policy information, and even geo-spatial data, as we and others [4,30] have done, is time-intensive and challenging. It is also necessary to fully understand a country’s progress towards their goals, and challenges requiring targeted inputs. Given that the neonatal period represents the disproportionate U5M mortality risk in most countries and in keeping with a life-course approach [31], there is an urgent need to accelerate improvements in antenatal care, midwifery, access to emergency obstetric care, and newborn resuscitation and care [32,33]. Concerns for quality of care, not merely coverage, were recurrent themes described by our key informants. A focus on quality, including workforce improvements, will be critical in the SDG period [34,35]. Finally, governance, leadership, and management were described by participants in our case studies and regional comparison as underdeveloped and often inadequately measured or tracked. Due to the lack of quantitative indicators or data for governance, leadership, and management, we utilized an approach combining qualitative data analyzed with a focus on codes related to the WHO’s definition with a national document review to assess these factors. Other studies have employed similar approaches, in addition to developing quantitative measures that ask key informants to rate governance and leadership in the systems they work in [36–38]. The importance of health governance and leadership is a recurring theme in global health that impacts MNCH care across health system levels, and one which requires novel approaches to measurement and intervention to accelerate SDG progress [39].

We believe much can be learned, not only from MDG successes and failures, but also from how such successes and failures are evaluated. Approaches combining ecological associations, nuanced qualitative research, and a policy review will be essential to guide SDG processes and priorities. Health information and data collection infrastructures need to be supported if consistent, comparable data are expected for tracking and reporting to evaluate SDG metrics.

## Supplementary Material

Supplemental Material

## Paper context

Millennium Development Goal 4 sought to reduce under-five mortality rates by two-thirds. This sparked many countries in Sub-Saharan Africa to adapt and scale-up international recommendations and interventions to improve child health, and efforts to evaluate progress towards this goal. We summarize work seeking to understand barriers and facilitators to child mortality in four Sub-Saharan African countries, and offer lessons and critiques for understanding progress in child health during the Sustainable Development Goal period.

## Supplementary material

The supplementary data for this article can be accessed here.
