# Supplementary material for: Measuring child survival for the Millennium Development Goals in Africa: what have we learned and what more is needed to evaluate the Sustainable Development Goals?
Source: Glob Health Action. 2020 Mar 2;13(1):1732668. doi: 10.1080/16549716.2020.1732668 (PMC7067162; doi:10.1080/16549716.2020.1732668)
Supplement: Supplemental Material [file ZGHA_A_1732668_SM7498.docx]

**Contents**

Table S1. Comparison of topic items explored in interviews with different groups of stakeholders.
Key Informant Interview Guide

Community Women Focus Group Discussion Guide

Table S1. Comparison of topic items explored in interviews with different groups of stakeholders.

|  | **MoH** | **Donor** | **NGO** | **Provider** |
| --- | --- | --- | --- | --- |
| **Topic 1: National Maternal Neonatal and Child Health Care System** |  |  |  |  |
| 1. strengths of the health care system | ✓ | ✓ | ✓ | ✓ |
| 1. weaknesses of the health care | ✓ | ✓ | ✓ | ✓ |
| 1. availability and distribution of MNCH services | ✓ | ✓ | ✓ | ✓ |
| 1. patient referral and follow-up | ✓ |  |  | ✓ |
| 1. communication of knowledge and best practices | ✓ | ✓ | ✓ | ✓ |
| 1. collaboration and sharing of resources and responsibility | ✓ | ✓ | ✓ | ✓ |
| 1. inequities within the National MNCH system | ✓ | ✓ | ✓ | ✓ |
| **Topic 2: National Maternal Neonatal Child Health Strategy** |  |  |  |  |
| 1. prevention and treatment needs at all levels of the health system | ✓ | ✓ |  |  |
| 1. prevention and treatment across the full continuum of MNCH Care | ✓ | ✓ |  |  |
| 1. effectively implemented at all levels of the health system | ✓ | ✓ |  |  |
| 1. National MNCH strategy changed over time? | ✓ | ✓ |  |  |
| **Topic 3: Maternal Neonatal and Child Health Interventions** |  |  |  |  |
| 1. most effectively implemented MNCH interventions | ✓ | ✓ | ✓ | ✓ |
| 1. least effectively implemented MNCH interventions | ✓ | ✓ | ✓ | ✓ |
| 1. acceptability and use of MNCH interventions by beneficiaries | ✓ | ✓ | ✓ | ✓ |
| **Topic 4: Clinical Standards and Guidelines Related to MNCH** |  |  |  |  |
| 1. clinical standards and guidelines with the most impact | ✓ |  |  | ✓ |
| 1. clinical standards and guidelines still needed | ✓ |  |  | ✓ |
| 1. clinical standards and guidelines changed over time | ✓ |  |  | ✓ |
| 1. awareness and use of appropriate clinical standards and guidelines | ✓ |  |  | ✓ |
| **Topic 5: Financial Flows and Human Resources Related to MNCH** |  |  |  |  |
| 1. MNCH funding is the strongest | ✓ | ✓ | ✓ |  |
| 1. Funding for MNCH is the weakest | ✓ | ✓ | ✓ |  |
| 1. MNCH funding changed over time | ✓ | ✓ | ✓ |  |
| 1. availability and quality of trained MNCH workers | ✓ |  | ✓ | ✓ |
| 1. Impact of untrained health care workers |  |  | ✓ | ✓ |
| **Topic 6: Effective Partnerships Related to MNCH** |  |  |  |  |
| 1. external partners helped improve child survival | ✓ | ✓ | ✓ | ✓ |
| 1. areas of MNCH *not* assisted by external partners | ✓ | ✓ | ✓ | ✓ |
| **Topic 7: Nutrition and MNCH** |  |  |  |  |
| 1. impact of malnutrition | ✓ | ✓ | ✓ | ✓ |
| 1. effective strategies to decrease malnutrition | ✓ | ✓ | ✓ | ✓ |
| 1. challenges in decreasing malnutrition | ✓ | ✓ | ✓ | ✓ |
| **Topic 8: Other Contextual Issues Related to MNCH** |  |  |  |  |
| 1. Internal conflict | ✓ | ✓ | ✓ | ✓ |
| 1. Drought or flooding | ✓ | ✓ | ✓ | ✓ |
| 1. Economy | ✓ | ✓ | ✓ | ✓ |
| 1. Government and political issues | ✓ | ✓ | ✓ | ✓ |

MoH, Ministry of Health; NGO, non-governmental organization; MNCH, maternal, neonatal, and child health

**Key Informant Interview Guide**

Topic 1: National Maternal Neonatal and Child Health Care System

*I would like to talk with you about the national Maternal, Neonatal, and Child Health system in this country and how it influences child survival*.

1. Can you describe the **strengths** of the health care system in this country with regards to prevention and treatment for maternal, neonatal and child health (MNCH)?
   1. [Probe: What is working well at each level of the MNCH health care system, including home/community services, primary care facilities, and regional or national referral centers?]
   2. [Probe: What is working well at the different points along the continuum of care including from pregnancy through delivery, the newborn period, the first year, and through young children up to 5 years?]
   3. [Probe: What has helped to make these parts of the MNCH health care system work well?]
2. Can you describe any **weaknesses** of the health care system in this country with regards to prevention and treatment of MNCH?
   1. [Probe: What does not work well at the home/community services level, primary care facilities level, and regional or national referral centers level?
   2. [Probe: What does not work well at the each point along the continuum of care from pregnant women through delivery, the newborn period, the first year, and through children up to 5 years?]
   3. [Probe: How can these weaknesses be overcome?]
3. How would you describe the **availability and distribution** of MNCH services in this country?
   1. [Probe: Do availability and distribution differ depending on whether those services are for prevention or for treatment?]
   2. [Probe: How available are MNCH services at each level of the MNCH health care system, including home/community services, primary care facilities, and regional or national referral centers?
   3. [Probe: How available are MNCH services at each point along the continuum of care from pregnant women through delivery, the newborn period, the first year, and through early childhood (5 years)?]
   4. [Probe: How would you describe the availability of essential MNCH-related medicines for both treatment and prevention?]
   5. [Probe: What is done when there is a shortage of essential MNCH-related medicines?]
   6. [Probe: What helped to make MNCH services available?]
   7. [Probe: What are the challenges in making MNCH services available and how can they be overcome?]
4. How would you describe **patient referral and follow-up** between the local community, primary care centers and higher-level referral centers?
   1. [Examples if needed (only provide if participant is having trouble answering the question): a pregnant woman needs referral from her local provider to a hospital for a cesarean section then returns home to continue care locally;
   2. [Examples if needed (only provide if participant is having trouble answering the question and the first example was not sufficiently helpful): a newborn requires referral to a regional hospital for intensive care at birth until they are stable for return to the community provider.
   3. [Probe: What are the challenges to patient referral and follow-up and how can they be overcome?]
5. Please describe how **knowledge and best practices related to child survival are communicated** *between* national health leaders, decision-makers, and implementers.)
6. [Probe: What could be done to improve communication between these groups?]
7. Please describe the **collaboration and sharing of resources and responsibility** between MNCH and this country’s *other* health sector programs, such as HIV/AIDS, malaria, immunization, nutrition, etc.
   1. [Example if needed (only provide if participant is having trouble answering the question): joint initiatives between MNCH and HIV/AIDS for preventing perinatal mother-to-child HIV transmission.]
   2. [Example if needed (only provide if participant is having trouble answering the question and the first example was not sufficiently helpful): collaboration between MNCH and the national malaria control programs to distribute bed nets or provide malaria treatment for fever.]
   3. [Probe: How effective is the collaboration and sharing of resources?]
8. Can you describe any **inequities** that may exist within the MNCH system in this country?
   1. [Examples if needed (only provide if participant is having trouble answering the question): is availability, access, or quality of prevention or treatment different depending on an individual’s wealth, area of residence, sex, education, or another factor?
   2. [Probe: How have inequities been successfully overcome?]
   3. [Probe: What are the challenges resulting in inequities and how can they be overcome?]

Topic 2: National Maternal Neonatal Child Health Strategy

*Now I would like to talk with you about your National MNCH strategic plan to improve child survival. The MNCH strategic plan refers to the overall priorities and written documents developed by the country’s health leadership that outlines how to achieve better MNCH.*

1. In your opinion, does the national MNCH strategic plan effectively address prevention and treatment needs **at all levels of the health system** in this country from the home/community, first-level health centers and referral centers? Please explain.
   1. [Probe: How could the national MNCH strategy be changed to improve child survival at any of the health care system levels from home/community, first-level health centers and referral centers?]
2. In your opinion, does the MNCH strategic plan adequately address prevention and treatment **across the full continuum of MNCH Care** from pregnancy and delivery, through the neonatal period, infancy, and childhood? Please explain.
   1. [Probe: How could the national MNCH strategy be changed to improve child survival at any of the points across the continuum from pregnancy and delivery to neonatal, infancy and childhood?]
3. Has the national MNCH strategic plan been **effectively implemented**?
   1. [Probe: Has the strategic plan been effectively implemented at each level of the MNCH health care system, including home/community services, primary care facilities, and regional or national referral centers?]
   2. [Probe: Has the strategic plan been effectively implemented at the different points along the continuum of care including from pregnancy through delivery, the newborn period, the first year, and through young children up to 5 years?]
   3. [Probe: What has helped make implementation of the strategic plan successful?
   4. [Probe: What are the challenges for successful implementation and how can they be overcome?
4. How have the **priorities** of the national MNCH strategic plan **changed over time**?

Topic 3: Maternal Neonatal and Child Health Interventions

*Now I would like to talk with you about specific MNCH interventions that are used to improve child survival in this country through either prevention or treatment of illness.*

1. Which MNCH interventions have **been** **most effectively implemented** in this country?
   1. [Probe: Which interventions have been effectively implemented at each level of the MNCH health care system, including home/community services, primary care facilities, and regional or national referral centers?]
   2. [Probe: Which interventions have been effectively implemented at the different points along the continuum of care including from pregnancy through delivery, the newborn period, the first year, and through young children up to 5 years?]
   3. [Probe: How would you describe the coverage of these interventions?]
   4. [Probe: How was effective implementation accomplished?]
   5. [Probe: Are these interventions sustainable? Please explain,]
2. Which MNCH interventions have **not been well implemented** in this country?
   1. [Probe: Which interventions have not been well implemented at each level of the MNCH health care system, including home/community services, primary care facilities, and regional or national referral centers?]
   2. [Probe: Which interventions have not been well implemented at the different points along the continuum of care including from pregnancy through delivery, the newborn period, the first year, and through young children up to 5 years?]
   3. [Probe: How would you describe the coverage of these interventions?]
   4. [Probe: What are the challenges to implementing these interventions and how can they be overcome?]
3. How would you describe the **acceptability and use** of MNCH interventions by pregnant women or their children in this country?
   1. [Probe: What has helped to make MNCH interventions acceptable and utilized?]
   2. [Probe: What are the challenges in making MNCH interventions acceptable and utilized to improve child survival?]

Topic 4: Clinical Guidelines Related to MNCH

*Now we will talk about clinical standards and guidelines that are nationally established to ensure standardized and effective prevention and treatment to improve child survival in this country.*

1. What clinical guidelines related to MNCH have had the **most impact** on child survival in this country?
   1. [Probe: Why have these guidelines had the most impact?]
2. What MNCH clinical guidelines **still need to be developed or implemented** in this country?
   1. [Probe: What are the challenges in developing or implementing these guidelines, and how can they be overcome?]
3. How have MNCH clinical guidelines in this country **changed over time**?
4. What should be done to improve the **awareness and use** of appropriate MNCH clinical guidelines in this country?
   1. [Probe: How can awareness and use of MNCH clinical guidelines be improved at each level of the MNCH health care system, including home/community services, primary care facilities, and regional or national referral centers?]
   2. [Probe: How can awareness and use of MNCH clinical guidelines be improved at the different points along the continuum of care including from pregnancy through delivery, the newborn period, the first year, and through young children up to 5 years?]

Topic 5: Financial Flows and Human Resources Related to MNCH

*Let’s discuss the resources this country currently has for MNCH, both financial and human resources for health, and how this affect child survival. We will be referring to prevention of child illness as well as treatment.*

1. Where do you think MNCH funding in this country is the **strongest** in terms of amount or sustainability or both?
   1. [Probe: Are funding strengths different at different levels of the health system including home/community, primary care centers, and referral centers?]
   2. [Probe: Are funding strengths different at different points along the continuum from pregnancy through delivery, newborns, infants, and young children up to 5 years?]
   3. [Probe: What has ensured these strong levels of funding?]
   4. [Probe: Are these strong levels of funding sustainable?]
2. Where do you think funding for MNCH in this country is the **weakest** in terms of amount or sustainability or both?
   1. [Probe: Are funding weaknesses different at different levels of the health system including home/community, primary care centers, and referral centers?]
   2. [Probe: Are funding weaknesses different at different points along the continuum from pregnancy through delivery, newborns, infants, and young children up to 5 years?]
   3. [Probe: What are the challenges for obtaining and sustaining funding for these areas and how can these be overcome?]
3. How has MNCH funding in this country changed over time?
4. How would you describe the availability and quality of trained MNCH workers in this country?
   1. [Probe: Are trained MNCH workers available at each level of the health system including home/community, first-level health centers, and referral centers?]
   2. [Probe: Are trained MNCH workers available at each point of the continuum of care from pregnancy through delivery, the newborn period, infancy, and through young children up to 5 years?]
   3. [Probe: How has availability and quality of trained MNCH workers changed over time?]
   4. [Probe: What helped to make trained MNCH workers available?]
   5. [Probe: What are the challenges in making trained MNCH workers available and how can they be overcome?]
   6. [Probe: Are trained community health workers a barrier to child survival or do they have an important role in improving MNCH?]

Topic 6: Effective Partnerships Related to MNCH

*The next questions relate to external partners, meaning international, national, regional or local partners not including government or health system leaders or workers, who may have influenced child survival in this country.*

1. How have external partners (e.g., Donors or implementing partners, NGOs, and others outside of the government or the national health system) helped improve child survival in this country?
   1. [Probe: How were effective external partnerships developed?]
2. What areas of MNCH in this country have *not* been assisted by external partners?
   1. [Probe: What types of external partnerships are most needed?]

Topic 7: Nutrition and MNCH

*I would like to discuss with you how nutrition and food availability have had an impact on MNCH in this country.*

1. How has food availability and nutrition had an impact on child survival in this country?
   1. [Probe: How has food availability and nutrition had an impact at each point of the continuum of care from pregnancy through delivery, the newborn period, infancy, and through young children up to 5 years?]
   2. [Probe: Is the impact of food availability and nutrition different depending on an individual’s wealth, area of residence, sex, education, or another factor? Please explain.]
2. What has helped to decrease malnutrition or increase food availability in this country?
3. What challenges exist to decreasing malnutrition or increasing food availability and how can these be overcome?

Topic 8: Other contextual issues affecting MNCH

*I would like to discuss with you how contextual factors other than nutrition have had an impact on MNCH in this country, such as internal conflict, drought or flooding, the economy, or government and politics.*

1. What impact, if any, has internal conflict had on child survival in this country? Please explain.
2. What impact, if any, has drought or flooding had on child survival in this country? Please explain.
3. What impact, if any, has the state of this country’s economy, have on child survival? Please explain.
4. What impact, if any, have governmental or political issues had on child survival? Please explain.

*At the end: Is there anything else that you would like to say about MNCH in this country, now or over the past 10 to 20 years?*

**Community Women Focus Group Discussion Guide**

Topic 1: Healthcare access and availability from pregnancy through early childhood

*For the first part of our discussion, we will talk about what happens when a woman needs healthcare during pregnancy to make sure her baby is born healthy, or when a young child needs healthcare (birth through age 5). We are only focusing on care for pregnant women and children.*

1. Let’s talk about when a woman is **pregnant or about to deliver her baby.**
   1. What type of healthcare might a pregnant woman need to improve the health of herself and her baby?
      1. [Examples to be used only if the respondents have difficulty responding. Do not provide all examples at once, and give respondents an opportunity to come up with their own answers: Routine care services during pregnancy and care for problems that may occur (e.g., fever, bleeding, baby not moving or growing as expected)?]
   2. What things help a woman get the healthcare she needs when she is pregnant or about to deliver her baby?
      1. [Examples to be used only if the respondents have difficulty responding. Do not provide all examples at once, and give respondents an opportunity to come up with their own answers: community support, available transportation, trust in healthcare workers/communication, quality of care provided, care is free, etc.]
   3. What makes it hard for a woman to get the healthcare she needs when she is pregnant or about to deliver her baby?
      1. [Examples: no healthcare in local community, cost, transportation, communication, quality, community or cultural norms/beliefs]
2. Now let’s talk about babies and young children, from newborn to five years old.
   1. What type of healthcare would someone need to improve the health of their babies or young children?
      1. If necessary, probe for newborns (birth to first month of life)
      2. If necessary, probe for infants (first month to first year of life)
      3. If necessary, probe for young children (up to five years old)
   2. What things help someone get the healthcare they need for their babies or young children?
      1. [Examples: community support, available transportation, trust in healthcare workers/communication, quality of care provided, care is free, etc.]
      2. If necessary, probe for newborns (birth to first month of life)
      3. If necessary, probe for infants (first month to first year of life)
      4. If necessary, probe for young children (up to five years old)
   3. What makes it hard for someone to get the care they need for their babies or young children?
      1. [Examples: no healthcare in local community, cost, transportation, communication, quality, community or cultural norms/beliefs]
      2. If necessary, probe for newborns (birth to first month of life)
      3. If necessary, probe for infants (first month to first year of life)
      4. If necessary, probe for young children (up to five years old)

Topic 2: Community factors that affect child health and survival

*For the second part of this discussion, we will talk about your community and the things here that affect women during pregnancy or a child’s health from birth through 5 years.*

1. Describe anything in your community that can affect a woman during pregnancy or a child’s health from birth through age 5?
   1. [Probe: Do sanitation and access to clean water impact the health of pregnant women or young children in your community? Please explain.]
   2. [Probe: Do nutrition and getting enough food impact the health of pregnant women or young children in your community? Please explain.]
   3. [Probe: Do community or cultural practices impact the health of pregnant women or young children in your community? Please explain.
      1. (Examples if needed: social support for new mothers, beneficial or harmful feeding practices, using local plants/herbs or other substances not provided by a trained health care worker to dry the cord, or using un-trained persons like traditional mid-wives or traditional healers for health care)]
   4. [Probe: Do health campaigns or programs impact the health of pregnant women or young children in your community? Please explain.]
   5. [Probe: Do droughts or floods impact the health of pregnant women or young children in your community? Please explain.]
   6. [Probe: Does internal conflict, government or economic instability impact the health of pregnant women or young children in your community? Please explain].
2. What do you feel has been done to improve these issues the group has discussed?
3. Is there anything else you think the **community, government, or health leaders should do** to address these issues and improve the health of pregnant women and young children from birth through age 5? Please explain.

Topic 3: Inequities in health care quality and availability

*For the final part of this discussion, we will talk about whether the quality and availability of health care is equal for all pregnant women and young children (birth through age 5) within the community.*

1. Within your community, are the same types and quality of **health services** available to all pregnant women or young children?
   1. [Probe if there are differences based on a person’s culture, religion, income, education, or sex, or based on where a person lives]
   2. [Probe whether the same types and quality of **health facilities** are available to all pregnant women or children]
   3. [Probe whether the same types and quality of **healthcare providers** are available to all pregnant women or children]
   4. [Probe whether the same types and quality of **medicines** are available to all pregnant women or children]
      1. **[**Examples of different health services to consider: healthcare during pregnancy and at birth; prevention of mother-to-child transmission of HIV; skilled attendant at delivery; management of newborns with difficulty breathing, low birth weight, or other conditions; immunizations; management of pneumonia, diarrhea, or malaria; etc.]

Topic 4: Other topics

*Before we conclude our discussion, is there anything else you think we should know about healthcare for pregnant women or children in this community? Please describe.*
